# Can Isoquinoline Alkaloids Affect Platelet Aggregation in Whole Human Blood?

**DOI:** 10.3390/toxins14070491

**Published:** 2022-07-15

**Authors:** Mst Shamima Parvin, Marcel Hrubša, Jaka Fadraersada, Alejandro Carazo, Jana Karlíčková, Lucie Cahlíková, Jakub Chlebek, Kateřina Macáková, Přemysl Mladěnka

**Affiliations:** 1 The Department of Pharmacognosy and Pharmaceutical Botany, Faculty of Pharmacy in Hradec Králové, Charles University, 500 05 Hradec Králové, Czech Republic; parvins@faf.cuni.cz (M.S.P.); karlickova@faf.cuni.cz (J.K.); cahlikova@faf.cuni.cz (L.C.); chlej2aa@faf.cuni.cz (J.C.); macakovak@faf.cuni.cz (K.M.); 2 The Department of Pharmacology and Toxicology, Faculty of Pharmacy in Hradec Králové, Charles University, 500 05 Hradec Králové, Czech Republic; hrubsam@faf.cuni.cz (M.H.); fadraerj@faf.cuni.cz (J.F.); carazofa@faf.cuni.cz (A.C.)

**Keywords:** isoquinoline, bulbocapnine, bleeding, antiplatelet, drug

## Abstract

Isoquinoline alkaloids have multiple biological activities, which might be associated with positive pharmacological effects as well as negative adverse reactions. As bleeding was suggested to be a side effect of the isoquinoline alkaloid berberine, we decided to ascertain if different isoquinoline alkaloids could influence hemocoagulation through the inhibition of either platelet aggregation or blood coagulation. Initially, a total of 14 compounds were screened for antiplatelet activity in whole human blood by impedance aggregometry. Eight of them demonstrated an antiplatelet effect against arachidonic acid-induced aggregation. Papaverine and bulbocapnine were the most potent compounds with biologically relevant IC_50_ values of 26.9 ± 12.2 μM and 30.7 ± 5.4 μM, respectively. Further testing with the same approach confirmed their antiplatelet effects by employing the most physiologically relevant inducer of platelet aggregation, collagen, and demonstrated that bulbocapnine acted at the level of thromboxane receptors. None of the alkaloids tested had an effect on blood coagulation measured by a mechanical coagulometer. In conclusion, the observed antiplatelet effects of isoquinoline alkaloids were found mostly at quite high concentrations, which means that their clinical impact is most likely low. Bulbocapnine was an exception. It proved to be a promising antiplatelet molecule, which may have biologically relevant effects.

## 1. Introduction

The class of isoquinoline alkaloids is one of the largest in the plant kingdom. As these alkaloids possess multiple pharmacological activities [1], several of them, such as morphine, codeine or tubocurarine, have already been used as clinically approved drugs. Many others have been tested in a row of animal in vivo studies and a few of them even reached clinical trials [2]. An example is berberine, which is especially known in traditional Chinese medicine; it could potentially be used as an antidiabetic and hypolipidemic drug. Berberine can, however, also cause gastrointestinal hemorrhage [2,3,4], which is not very surprising given its known antiplatelet effects. Other isoquinoline alkaloids have also previously been shown to block platelet aggregation; on the other hand, there are no clear data on whether these effects have a clinical significance. Such effects could be useful in ischemic cardiovascular diseases, but might be associated with adverse bleeding when administered for other purposes. Antiplatelet properties were reported for a series of isoquinoline alkaloids including allocryptopine, berberine, boldine, glaucine, isocorydine, papaverine, protopine and scoulerine. These data, however, mostly originate from experiments with purified platelets or platelet-rich plasma (PRP) isolated from the blood of healthy donors or animals [5,6,7,8,9,10,11]; moreover, many studies were purely screening studies and reported an inhibition only at one or a few very high concentrations such as 100 μM or 100 μg/mL (approximately 200–300 μM) [6,8,11]. Berberine is an exception; its potential antiplatelet effects were confirmed in vivo in rats and other data reported that it was also active in purified platelets sensitized by hyperglycemia [5,9]. One study found a strong antiplatelet effect of the alkaloid protopine, which was confirmed in animals using arachidonic acid (AA) as a platelet activation trigger; another study found a much weaker effect of this alkaloid toward AA [6,10]. On the other hand, it should also be mentioned that there is one report suggesting that berberine can have the opposite effect as it can promote thrombosis through the facilitation of blood coagulation [12].

**Figure 1:**
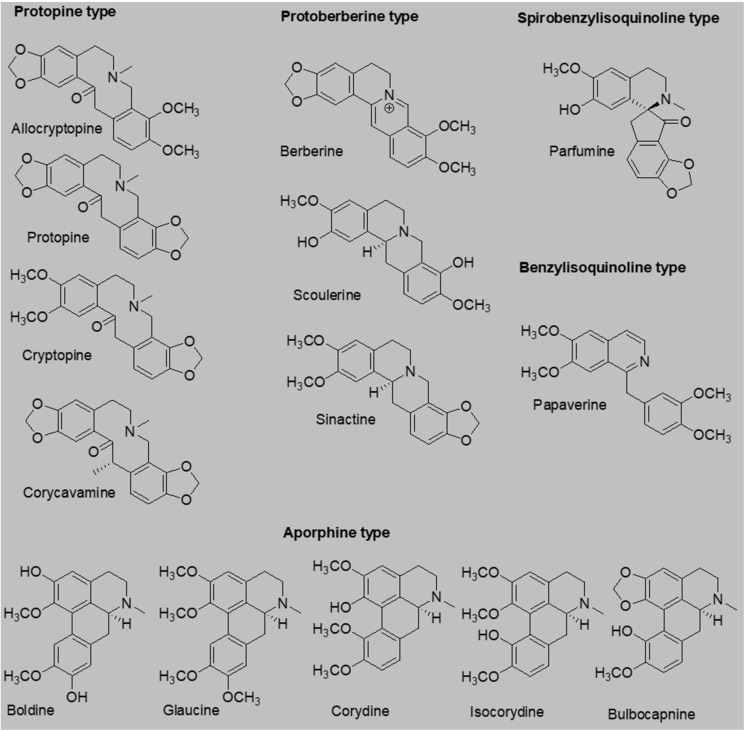
Structures of studied isoquinoline alkaloids.

Another interesting aspect is the fact that many isoquinoline alkaloids also have cytostatic effects [1,13,14,15]. Tumors have been shown to promote platelet aggregation and trigger thrombotic episodes also known as tumor cell-induced platelet aggregation whilst platelets can promote cancer growth and metastasis [16,17]. Taking this discovered link between platelets and cancer into account, antiplatelet effects might be useful in cancer treatments. The published studies with isoquinoline alkaloids documented a variable potency (IC_50_) against different cancer cell lines, ranging from 3 μM to almost inactive compounds [1,13,14,15,18]. Based on the aforementioned information, we decided to test the antiplatelet effects of a palette of 14 isoquinoline alkaloids (Figure 1), 6 of which, as far as we know, have never been tested for their antiplatelet effects.

Moreover, advances in this area also enabled the testing of platelet aggregation in whole blood, which is particularly relevant because other cells present in the blood can also impact the whole process. The coagulation of human plasma was included in the investigation as well. In addition, we aimed to decipher the mechanism of action of the most potent compound.

## 2. Results

**Figure 2:**
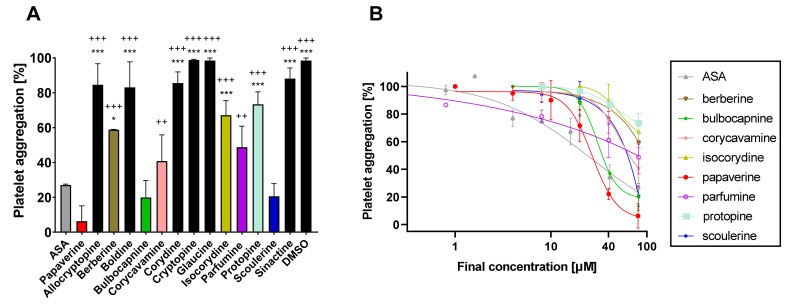
Antiplatelet effect against platelet aggregation triggered by arachidonic acid. (**A**) Comparison at a final concentration of 80 μM. (**B**) Concentration-dependent curves of the active compounds. The colors correspond with part (**A**) of this figure. Compounds with black columns were inactive (vs. DMSO, the negative control). * *p* < 0.05 vs. ASA (acetylsalicylic acid, positive control); *** *p* < 0.001 vs. ASA; ++ *p* < 0.05 vs. papaverine; +++ *p* < 0.001 vs. papaverine.

Fourteen isoquinoline alkaloids including well-known antiplatelet compounds berberine and papaverine were screened for their possible antiplatelet effects in the first step. Initial screening with a final concentration of 80 μM of the tested compounds showed that eight of them were able to block platelet aggregation induced by AA in whole blood (Figure 2A). Papaverine was numerically the most potent, but bulbocapnine, scoulerine, corycavamine and parfumine demonstrated comparable effects with the standard antiplatelet drug acetylsalicylic acid (ASA) according to the statistical analysis. Therefore, concentration–antiplatelet activity curves for all eight active alkaloids were constructed (Figure 2B). The most potent compound, papaverine, had an IC_50_ of 26.9 ± 12.2 μM followed by bulbocapnine with an IC_50_ of 30.7 ± 5.4 μM. The IC_50_ of the clinical standard ASA was 23.8 ± 10.4 μM [19]. Other alkaloids were clearly less active with IC_50_ values > 50 μM.

**Figure 3:**
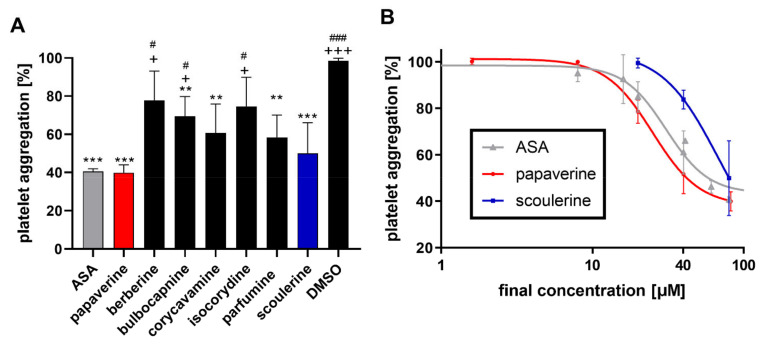
Effect of active alkaloids on collagen-triggered platelet aggregation. (**A**) Comparison of active alkaloids at a concentration of 80 μM. (**B**) Concentration-dependent curves of the most active compounds. ** *p* < 0.01 vs. the negative control (DMSO); *** *p* < 0.01 vs. DMSO; + *p* < 0.05 vs. ASA (acetylsalicylic acid); +++ *p* < 0.001 vs. ASA; # *p* < 0.05 vs. papaverine; ### *p* < 0.001 vs. papaverine.

Given the weak effect of protopine, this compound was omitted from further testing whilst the remaining seven compounds were used for further screening with a more physiological aggregation inducer, collagen, to confirm their anti-aggregation potential (Figure 3A). Only 5 of the tested alkaloids were active in this setting at the same screening final concentration of 80 μM. Interestingly, scoulerine was more active than bulbocapnine with this inducer. On the other hand, isocorydine and berberine were not active. Concentration–antiplatelet activity curves were constructed for papaverine and scoulerine (Figure 3B).

**Figure 4:**
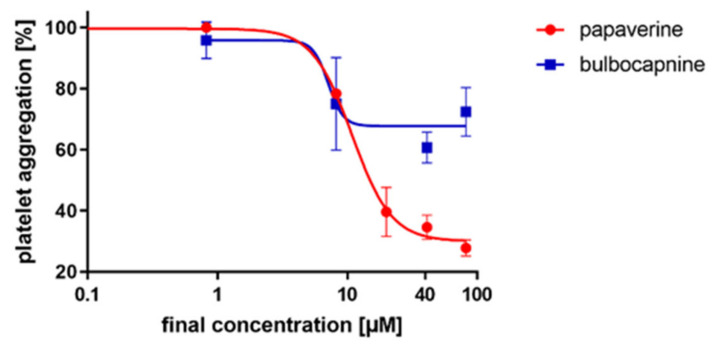
Antiplatelet effect of papaverine and bulbocapnine on thromboxane analog U46619-induced aggregation. Whole human blood was incubated with a solvent (DMSO) or different concentrations of tested compounds. U46619 at a final concentration of 130 nM was then added to induce the platelet aggregation.

In the next step, we focused on the AA-based cascade where papaverine and bulbocapnine showed comparable IC_50_ values. The mechanism of papaverine is known as it is a non-selective inhibitor of phosphodiesterases [20]. Hence, only the mechanism of action of bulbocapnine was further investigated. In the AA aggregation cascade, AA is initially transformed by cyclooxygenase 1 into prostaglandin H_2_, which is subsequently converted into thromboxane A_2_ by thromboxane synthase. However, sensitive ELISA assays did not demonstrate any inhibition of these enzymes by bulbocapnine (data not shown). Thromboxane A_2_ is known to induce platelet aggregation via its Gq receptors. However, as it is a highly unstable compound, its stable analog U46619 is used in experimental settings. Bulbocapnine was partly able to block the platelet aggregation induced by this pathway. Papaverine was tested for a comparison as it stabilizes platelets via an increase in cAMP [21,22]. As expected, papaverine was more active. Based on these results, we supposed that bulbocapnine could likely act as a partial antagonist at the thromboxane receptors (Figure 4).

**Figure 5:**
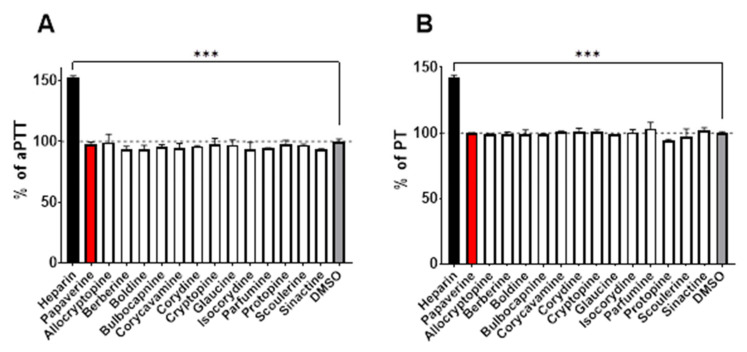
Effect of the tested compounds on blood coagulation. Comparison of activated partial thromboplastin time (aPTT) values (**A**) and prothrombin time (PT) values (**B**) of tested compounds at a final concentration of 100 μM. DMSO was used as a vehicle at a final concentration of 1% and heparin was used as a positive control at final concentrations of 5 IU/mL and 0.5 IU/mL for PT and aPTT tests, respectively. *** *p* < 0.001 vs. solvent (DMSO).

In the last step, we also tested whether these alkaloids could affect the coagulation cascade as in a few circumstances both antiplatelet drugs and anticoagulant drugs are used. None of the compounds were active either on the prothrombin time (PT) or the activated partial thromboplastin time (aPTT; Figure 5).

## 3. Discussion

Several isoquinoline alkaloids have previously been tested for their platelet aggregation so their data can be at least partially compared. In addition to the well-known potent effects of papaverine [6], berberine is one of the most tested compounds. A recent study reported that its IC_50_ on collagen-triggered aggregation in purified human platelets was 33 μM and 21 μM on thrombin-induced aggregation [5]. Such data appeared to be promising, but there are a few major problems related to the possible clinical use of berberine. The first major issue are the potential side effects; in particular, the risk of hepatotoxicity and cytotoxic effects [1,3]. Secondly, berberine is a quaternary ammonium salt with a very low oral bioavailability [4]; hence, the tens of μM plasma concentrations that would be required to observe this activity are not achievable with an oral administration. Importantly, our study showed that much higher concentrations were required for the effective inhibition of platelet aggregation in whole blood. We did not precisely establish the IC_50_ as berberine was only weakly active. At a concentration of 80 μM, it blocked the platelet aggregation triggered by AA by approximately 40% and even less in the case of collagen (Figure 2 and Figure 3). This indicated that the IC_50_ was clearly higher than 80 μM in both cases. The difference between the results from purified platelets and whole blood suggests that berberine binds to plasma proteins or other blood cells, which decreases the amount of available berberine for the inhibition of the platelets. It should also be mentioned that the effect of berberine was weak on purified rabbit platelets where only a moderate inhibition of 10–30% was observed with a high concentration of berberine (either as chloride or iodide salt) using a series of different inducers including ADP, AA, collagen and platelet-activating factor (PAF) [6]. On the other hand, the antiplatelet effects of berberine were confirmed in vivo in rats, but berberine was given i.v. in relatively high doses ranging from 10 to 30 mg/kg in that study [9]. Interestingly, in the same study, the inhibition of the formation of thromboxane A_2_ was accompanied by an inhibition of prostacyclin synthesis. This might be another important factor hindering the potential use of berberine in cardiovascular indications as an inhibition of prostacyclin synthesis is undesirable [23].

More data are also available on protopine. It fully blocked the collagen- and PAF-triggered aggregation of purified rabbit platelets at a concentration of 100 μM, but had only about a 50% effect on AA and 16% on ADP [6]. This finding is in contrast with another study, in which PAF-induced rabbit platelet aggregation was blocked only by 28% at a concentration of 100 μg/mL (283 μM). There was a small inhibition of collagen-based aggregation (~14%) at a concentration of 20 μg/mL (57 μM) [11]. The results from this study confirmed the mild inhibition of AA-triggered platelet aggregation (~30%) at a similarly high concentration of 80 μM, but showed that the effect rapidly dropped to zero when decreasing the concentrations (Figure 2A, B). It must be mentioned that there are also other data on protopine that showed a stronger antiplatelet effect in human PRP induced to aggregate by four different inducers (AA, PAF, collagen and ADP) with IC_50_ values ranging from 9 to 16 μM [10]. Such results are at a strong contrast with our and other results. As the concentrations of the inducers were clearly much higher in that study (800 μM of AA and 20 μg/mL of collagen) than in our study, rather lower inhibitory effects of protopine were expected. There were only two other apparent differences; they used PRP vs. whole blood and citrate as the anticoagulant vs. heparin in our study. The authors also confirmed the protective effect of protopine on AA- or PAF-induced thrombosis in an in vivo model in rabbits, but the doses were very high (50–100 mg/kg, i.p.). Such large doses could not be administered to humans.

Data on allocryptopine from washed rabbit platelets agreed with our results as only a mild inhibition (12–22%) was observed for ADP-, AA-, collagen- and PAF-induced aggregation at a concentration of 100 μM [6] vs. 17% with AA in this study at a concentration of 80 μM (Figure 2A). Glaucine was inactive in our study as well as in the study of Chen et al., which employed washed rabbit platelets [8]. The same study also reported a strong effect of boldine, which fully abolished AA-based aggregation and markedly decreased that of collagen. That study, however, employed boldine at a concentration of 100 μg/mL (269 μM). Apparently, its effects markedly dropped with lower concentrations (Figure 2A). In the aforementioned study [8], isocorydine also had an antiplatelet potential, which was confirmed in this study with both AA and collagen.

Six of the alkaloids we isolated (corydine, corycavamine, cryptopine, sinactine and parfumine) were, as far as we know, tested for the first time for their antiplatelet activity. Importantly, two of them, corycavamine and parfumine, were among the most active (Figure 2 and Figure 3). Both compounds had a good effect at a concentration of 80 μM, but the effect dropped and disappeared at lower concentrations. We did not test their effects and mechanisms further due to the necessity of using high concentrations, which exclude a possible biological relevance. It is, however, possible that a chemical derivation, in particular that of parfumine (the only tested spirobenzylisoquinoline), could improve the effect.

Out of all the tested compounds, bulbocapnine appeared to be the best candidate for a potential antiplatelet compound. It does not bind to topoisomerases as its aporphine congener dicentrine [24] or previously mentioned berberine [25,26] do, so cytostatic effects are not expected. It also possesses other cardiovascular effects such as peripheral vasodilation [27]. Bulbocapnine is, therefore, potentially useful as a cardiovascular drug, but might cause bleeding, as is the case with all other clinically or experimentally confirmed antiplatelet compounds. Its potency was lower than clinically used ASA, but it apparently had a different mechanism of action. ASA inhibits cyclooxygenase 1, but bulbocapnine had no effect on this enzyme. Its effects were partly mediated by antagonism at the thromboxane receptors. Given the known high resistance of some patients—in particular, diabetic patients—to ASA [28], a drug with a different mechanism overpassing this resistance is urgently needed. In order to test the relevance, pharmacokinetic testing of bulbocapnine—in particular, in relation to the feasibility of its oral administration—must be performed. Another interesting aspect is its distinctive methylenedioxy bridge, which the other tested aporphine alkaloids do not have. This structural feature is also present in other active isoquinolines (e.g., berberine, corycavamine and protopine). This group seems to be advantageous for antiplatelet effects, but do not assure an antiplatelet effect (e.g., cryptopine and allocryptopine).

Platelets have also been shown to play a role in cancer development [16,17]; for this reason, a compound with both antiplatelet effects and cytostatic activity could be of potential therapeutic use. From our study, scoulerine appeared to be a good candidate as its anticancer effects were observed in various cancer cell lines with IC_50_ values ranging from 3 to 6 μM [14,18]. Scoulerine is an interesting compound as it has a better effect on collagen- than AA-based platelet aggregation; this was found previously [11] with high concentrations and confirmed in lower concentrations in this study (Figure 2 and Figure 3). 

Another aspect is the potential impact of the tested compounds on blood coagulation. This derives from previous studies with berberine, which was shown to both: (1) increase the expression of the tissue factor both in vitro and in vivo, which can be associated with pro-coagulation [12]; and (2) decrease the expression of the tissue factor [29]. Therefore, we also performed a screening coagulation test with all the included alkaloids. With regard to aPTT and PT values (Figure 5), none of the tested alkaloids affected the blood coagulation ex vivo.

## 4. Conclusions

This study showed that the tested isoquinoline alkaloids were not likely to cause bleeding due to their rather weak antiplatelet effects and no effect on coagulation. We observed significant effects on platelet aggregation at relatively high concentrations, which are probably not achievable even if non-quaternary ammonium isoquinolines fulfill the theoretical requirements for oral absorption [1]. A possible exception is bulbocapnine. Its distinct structural feature coupled with its relatively high activity certainly warrants further exploration because it has a different mechanism of action from current clinically used antiplatelet drugs. Therefore, it could be developed as an alternative drug in cases of resistance and/or for combinations with other antiplatelet drugs. However, its pharmacokinetics, with an emphasis on achievable plasma levels, should be firstly investigated. Of interest is that many active isoquinoline alkaloids possess a methylendioxy group and this can be a base for the modification of these alkaloids in a search for more potent antiplatelet congeners. From another point of view, intoxication with *Corydalis* and *Dicentra* species containing bulbocapnine might have an impact on platelet aggregation and could, therefore, potentially cause bleeding.

## 5. Materials and Methods

### 5.1. Reagents

ASA, indomethacin, ethylenediaminetetraacetic acid (EDTA), 1-benzylimidazole and terutroban were purchased from Sigma Aldrich (Prague, Czech Republic). U-46619, a thromboxane B_2_ ELISA kit, prostaglandin H_2_ and a cyclooxygenase (COX) inhibitor screening assay kit were obtained from the Cayman Chemical Company (Ann Arbor, MI, USA). Collagen was purchased from Diagnostica a.s. (Prague, Czech Republic) and heparin sodium from Zentiva (Prague, Czech Republic). AA was obtained from Roche (Prague, Czech Republic) and sodium chloride (0.9%) from B. Braun (Prague, Czech Republic); dimethyl sulfoxide (DMSO) and 96% ethanol were bought from Penta (Prague, Czech Republic). Human control plasma, prothrombin time reagent (Technoplastin^®^ HIS), activated partial thromboplastin time (aPTT) reagent (DAPTTIN^®^) and calcium chloride were purchased from Technoclone (Vienna, Austria).

### 5.2. Tested Compounds

Berberine chloride, isocorydine and papaverine hydrochloride were purchased from Sigma Aldrich (Prague, Czech Republic), glaucine was purchased from the Cayman Chemical Company (Ann Arbor, MI, USA) and boldine was purchased from Carl Roth (Karlsruhe, Germany). All other alkaloids were isolated by members of the ADINACO research group at the Department of Pharmacognosy and Pharmaceutical Botany, Faculty of Pharmacy, in Hradec Králové, Charles University, Czech Republic. Bulbocapnine, corycavamine and corydine were isolated from *Corydalis cava* [30], scoulerine from *Eschscholzia californica* [31], cryptopine, parfumine and sinactine from *Fumaria officinalis* [32] and allocryptopine and protopine from *Chelidonium majus* [33].

### 5.3. Volunteers and Blood Preparation

Blood samples were taken from non-smoking healthy volunteers by venipuncture into plastic disposable tubes coated with heparin sodium (17 IU/mL). For the measurements performed with an impedance aggregometer, whole blood was used. In the case of a thromboxane synthase assay, indomethacin (COX inhibitor) was immediately added to the collected blood at a final concentration of 10 μM. The whole blood was centrifuged for 8 min at 214 g (centrifuge VWR Compact Star CS4, VWR International Ltd., Poole, UK); PRP was obtained as a supernatant solution. Platelet-poor plasma was isolated from the remaining blood by centrifuging for 10 min at 2500 g and was used for an adjustment of the PRP to 3.5 × 10^8^ platelets per mL. A Neubauer Improved counting chamber (Marienfeld, Lauda-Königshofen, Germany) and an inverted Nikon Eclipse TS100 microscope (Nikon Corporation, Tokyo, Japan) were used for platelet counting. All volunteers signed written informed consent. The study was approved by the Ethics Committee of Charles University, Faculty of Pharmacy, in Hradec Králové (approval date: 12 November 2012) and conformed with the latest Declaration of Helsinki.

### 5.4. Inhibition of Arachidonic Acid and Collagen Induces Platelet Aggregation

An initial screening of the antiplatelet effects was performed with whole human blood using an impedance aggregometer Multiplate^®^ (Roche, Switzerland) [34]. The detailed measurement procedure has been previously described [19]. In brief, 300 μL of whole blood was diluted with an equivalent amount of preheated 0.9% sodium chloride and incubated for 3 min at 37 °C with 5 μL of the appropriate tested compound (dissolved in DMSO, final concentration < 1%) or DMSO (blank sample). The aggregation reaction was initiated by the addition of AA or collagen and recorded for 6 min. The final concentration range of AA and collagen was 32–147 μM and 0.3–1.6 μg/mL, respectively, according to a double-calibration procedure. ASA was employed as the standard for both AA- and collagen-induced aggregation.

### 5.5. Cyclooxygenase-1 Inhibition

A commercial set from Cayman Chemical Company was used for the ELISA assessment of COX-1 inhibition [35]. The tested compounds were incubated with ovine COX-1 at 37 °C and AA at a final concentration of 100 μM. ASA was used as a standard COX-1 inhibitor. The formed prostaglandin H_2_ was measured following its reduction to prostaglandin F_2α_ by stannous chloride. The percentage of inhibition was related to the positive control containing only the solvent and AA.

### 5.6. Thromboxane Synthase Inhibition

The PRP was incubated with a tested compound for 3 min at 37 °C. After that, 50 ng of prostaglandin H_2_ was added into the mixture and incubated precisely for an additional 5 min. The incubation was then ended by adding 2 mM of a solution of chilled EDTA. A commercial kit from the Cayman Chemical Company was used for the determination of the thromboxane B2 levels in the supernatant [36]. The data were compared with 1-benzylimidazole, a known inhibitor of thromboxane A_2_ synthase.

### 5.7. Antagonism at the Thromboxane Receptors

To determine the antagonism at the thromboxane receptor, the same procedure as in the case of AA was used, but the aggregation was induced by the addition of U-46619, a stable agonist at the thromboxane receptor. The results were compared with a standard substance, epicatechin. The final concentration of U-46619 was 130 nM. A standard turbidimetric method with a Chrono-log 500-Ca aggregometer (Chrono-log Co., Havertown, PA, USA) connected to a computer (Aggro/Link software, Chrono-log Co.) was used for the confirmation of the obtained data [37].

### 5.8. Anticoagulant Experiments

Commercially available normal human control plasma (Technoclone, Vienna, Austria) was used for the anticoagulant experiments in a Ceveron^®^ Four coagulometer (Technoclone). DMSO (final concentration of 1%) or DMSO solutions of the tested compounds were incubated with plasma (100 μL) at 37 °C. The incubation time was 1 min for the PT assay, after which 200 μL of TECHNOPLASTIN-HIS was added and the time was recorded. For the aPTT assay, the treated control plasma was incubated with 100 μL of DAPTTIN and incubated for 2 min prior to the addition of 100 μL of 25 mM CaCl_2_ and the time was recorded. For every assay, positive controls using heparin (5 IU/mL and 0.5 IU/mL in the PT and aPTT assays, respectively) were included.

### 5.9. Statistical Analysis

Data were presented as means ± SD. The differences between the compounds in the antiplatelet and coagulation assays were assessed by a one-way ANOVA followed by the Tukey multiple comparison test. The differences between the concentration response curves and the reduction lines were analyzed using 95% confidence intervals. The entire statistical evaluation was carried out using GraphPad Prism 9.3.1 (GraphPad Software, San Diego, CA, USA).
